# Deficiency of *Wdr60* and *Wdr34* cause distinct neural tube malformation phenotypes in early embryos

**DOI:** 10.3389/fcell.2023.1084245

**Published:** 2023-05-09

**Authors:** Lu Yan, Hailing Yin, Yiwei Mi, Yu Wu, Yufang Zheng

**Affiliations:** ^1^ Obstetrics and Gynecology Hospital, The Institute of Obstetrics and Gynecology, Fudan University, Shanghai, China; ^2^ Department of Cellular and Developmental Biology, School of Life Sciences, Fudan University, Shanghai, China; ^3^ State Key Laboratory of Genetic Engineering, School of Life Sciences, Fudan University, Shanghai, China; ^4^ Obstetrics Department of the First Affiliated Hospital with Nanjing Medical University, Nanjing, Jiangsu, China

**Keywords:** *Wdr60*, *WDR34*, cilia, SHH signaling, PCP signaling

## Abstract

Cilia are specialized organelles that extend from plasma membrane, functioning as antennas for signal transduction and are involved in embryonic morphogenesis. Dysfunction of cilia lead to many developmental defects, including neural tube defects (NTDs). Heterodimer *WDR60*-*WDR34* (WD repeat domain 60 and 34) are intermediate chains of motor protein dynein-2, which play important roles in ciliary retrograde transport. It has been reported that disruption of *Wdr34* in mouse model results in NTDs and defects of Sonic Hedgehog (SHH) signaling. However, no *Wdr60* deficiency mouse model has been reported yet. In this study, piggyBac (PB) transposon is used to interfere *Wdr60* and *Wdr34* expression respectively to establish *Wdr60* ^*PB/PB*^ and *Wdr34* ^*PB/PB*^ mouse models. We found that the expression of *Wdr60* or *Wdr34* is significantly decreased in the homozygote mice. *Wdr60* homozygote mice die around E13.5 to E14.5, while *Wdr34* homozygote mice die around E10.5 to E11.5. *WDR60* is highly expressed in the head region at E10.5 and *Wdr60* ^*PB/PB*^ embryos have head malformation. RNAseq and qRT-PCR experiments revealed that Sonic Hedgehog signaling is also downregulated in *Wdr60* ^*PB/PB*^ head tissue, demonstrating that WDR60 is also required for promoting SHH signaling. Further experiments on mouse embryos also revealed that the expression levels of planar cell polarity (PCP) components such as CELSR1 and downstream signal molecule c-Jun were downregulated in *WDR34* homozygotes compared to wildtype littermates. Coincidently, we observed much higher ratio of open cranial and caudal neural tube in *Wdr34* ^*PB/PB*^ mice. CO-IP experiment showed that *WDR60* and *WDR34* both interact with IFT88, but only *WDR34* interacts with IFT140. Taken together, *WDR60* and *WDR34* play overlapped and distinct functions in modulating neural tube development.

## Introduction

Primary cilia are hair-like organelles protruding from cellular surface. They exist on various tissue, playing vital roles in embryogenesis as they mediate essential signaling pathways such as Hedgehog (HH) and WNT signaling (Eggenschwiler and Anderson, 2007; Goetz and Anderson, 2010; Anvarian et al., 2019). Defects in cilia genes can cause severe birth defects called ciliopathies, which affect multiple organs. Typical symptoms of ciliopathies include polydactyly, situs inversus, polycystic kidneys, etc (Hildebrandt et al., 2011; Reiter and Leroux, 2017). Mutations in some cilia genes have also been associated with human neural tube defects (NTDs) (Lei et al., 2015; Miao et al., 2016; Hofmeister et al., 2018; Shi et al., 2018; Kim et al., 2019; Yin et al., 2020), which we summarized in a recent review (Yan and Zheng, 2022). NTDs are severe structural birth defects resulted from a failure of neural tube closure. Based on different regions of unclosed neural tube, NTDs have multiple clinical features including anencephaly, exencephaly, myelomeningocele, and spina bifida (Detrait et al., 2005; Robinson et al., 2012; Wallingford et al., 2013).

Though tiny in size, the inner structure of cilia is sophisticate, comprising five major parts, which are basal body, transition zone, axoneme, intraflagellar transport (IFT) complex, and ciliary membrane (Sharma et al., 2008). The construction and function of cilia depends on IFT system and microtubule-based motor proteins inside cilia. The IFT complexes, composed of IFTA and IFTB subunits, move along the axonemal microtubules to transport cargos between the base and tip of cilia. Kinesin-2 is the anterograde motor protein to move IFT complexes from the base to the tip of cilia; while cytoplasmic dynein-2 is the retrograde motor protein to move IFT complexes from the tip to the base of cilia (He et al., 2017; Wang and Dynlacht, 2018; Anvarian et al., 2019).

Cytoplasmic dynein-2 complex contains multiple proteins, including the cytoplasmic dynein-2 heavy chains, light chains, intermediate chains, and light intermediate chains (He et al., 2017; Roberts, 2018). To be noticed, ciliary dynein-2 adopts an asymmetric conformation, which makes it distinguished from cytoplasmic dynein-1 in structure (Roberts, 2018; Toropova et al., 2019); and the asymmetry of dynein-2 is resulted from the WDR60-WDR34 heterodimer, which is the only heterodimer in dynein-2 complex (Toropova et al., 2019). WDR60 (WD repeat domain 60) and WDR34 (WD repeat domain 34) are two dynein-2 intermediate chain proteins (Asante et al., 2014; Tsurumi et al., 2019). Defects in *WDR60* gene have been associated with ciliopathies such as short rib-polydactyly syndrome and Jeune asphyxiating thoracic dystrophy (McInerney-Leo et al., 2013; Cossu et al., 2016; Kakar et al., 2018); and defects in *WDR34* gene have been associated with ciliopathies such as short-rib thoracic dysplasia 11 with or without polydactyly (Huber et al., 2013; Schmidts et al., 2013). Overlapping functions of WDR60 and WDR34 are verified in previous studies, including ciliary retrograde transportation, stabilization of transition zone, and ciliary gating (Hamada et al., 2018; Vuolo et al., 2018; Tsurumi et al., 2019; De-Castro et al., 2022). Despite of these, distinctive functions of WDR60 and WDR34 have also been reported in cell based study. WDR60 but not WDR34 is responsible for dynein-2 assembly; while WDR34 but not WDR60 is essential for axonemal extension (Vuolo et al., 2018).

Previously we reported that mutations in *WDR34* are associated with human NTD (Yin et al., 2020). Coincidently, the reported *Wdr34* ^*−/−*^ mutant mice also exhibit exencephaly (Wu et al., 2017). However, there is no report of WDR60 deficiency in animal models yet. Here we used the piggyBac (PB) transposon system (Ding et al., 2005) to establish *Wdr60* and *Wdr34* mouse mutants and explored the potential function of WDR60 in embryonic development. Deficiency of *Wdr60* elicits abnormal head development at E10.5 and multiple ciliopathy phenotypes and holoprosencephaly at E12.5 to E13.5. All *Wdr60* ^*PB/PB*^ homozygote die before E14.5, while *Wdr34* homozygote mice die around E10.5 to E11.5. Similar to *WDR34*, loss of *WDR60* also leads to decreased SHH signaling and dorsalized neural tube patterning. However, qRT-PCR and immunofluorescent experiments revealed that WDR34 has more effects on planar cell polarity (PCP) pathway than WDR60. Coincidently, we observed much higher penetrance of open cranial and caudal neural tube in *Wdr34* ^*PB/PB*^ mice. We further verified both WDR60 and WDR34 interact with IFT88, but only WDR34 interacts with IFT140. Therefore, WDR60 and WDR34 play overlapped and distinct functions in modulating head neural tube development.

## Results

### Disruption of WDR60 and WDR34 in mouse models

**FIGURE 1:**
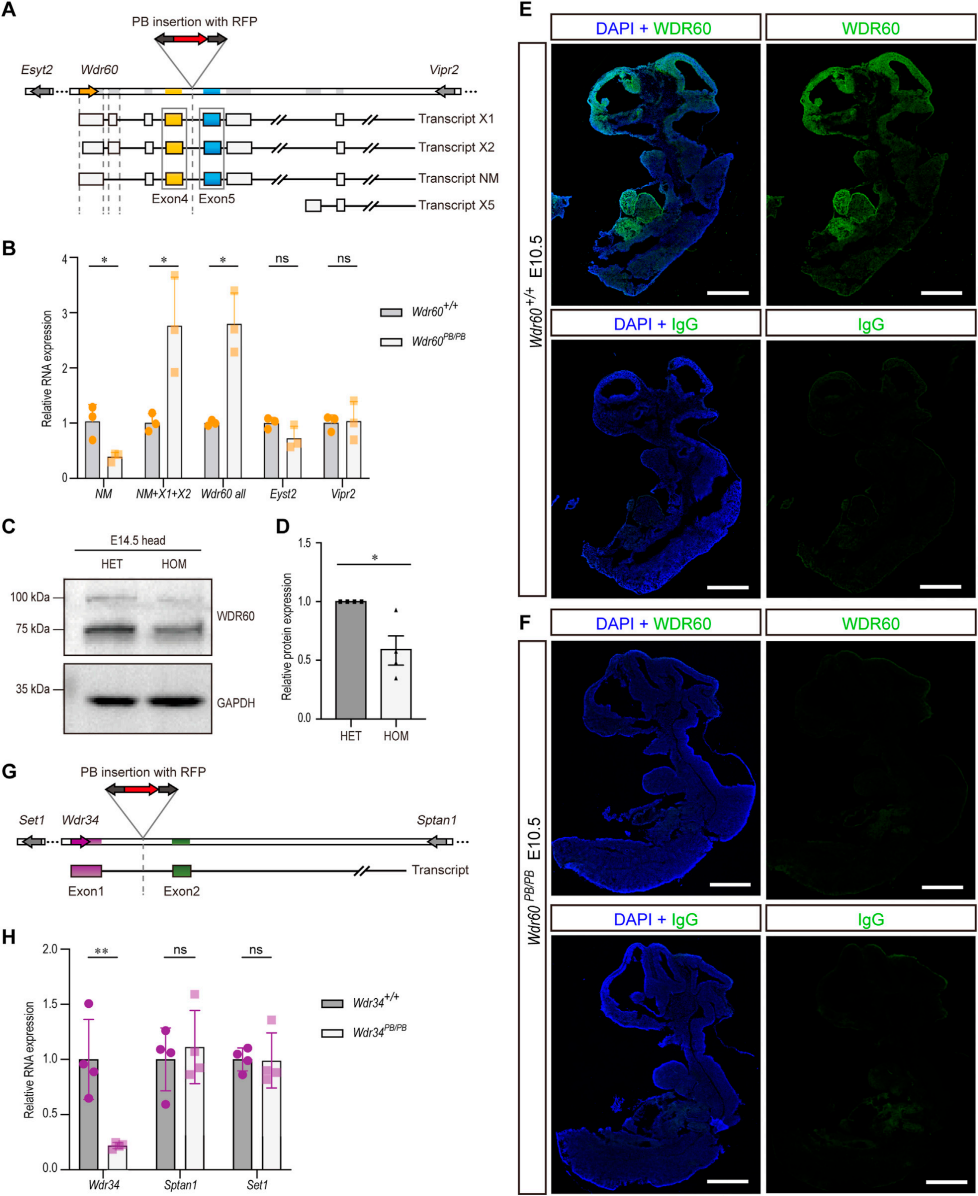
Disruption of *Wdr60* and *Wdr34* expression in mouse by *PB* transposon. **(A)** Schematic *Wdr60* gene and different transcripts with PB insertion. There are four predicted transcriptional variants for *Wdr60*, which are indicated as transcript X1, X2, NM (NM_146,039.3), and X5. **(B)** Transcriptional expression levels of *Wdr60* transcripts (NM alone, NM + X1+X2, and all transcripts) and the nearby genes *Eyst2* and *Vipr2* were measured by qRT-PCR in *Wdr60* ^*PB/PB*^ and WT (*Wdr60* ^*+/+*^) littermates. (N = 3 litters, at least 2 embryos per genotype were used in each litter) **(C)** E14.5 embryo heads were collected for both *Wdr60* ^*+/PB*^ (HET) and *Wdr60* ^*PB/PB*^ (HOM) embryos. Total proteins were extracted and immunoblots with anti-WDR60 antibody. The band at around 100 kDa is much less in HOM embryos than that in HET embryos and the quantification is provided in **(D)**. **(E)** Sagittal sections of E10.5 WT embryos were used for immunofluorescent experiment and WDR60 (green) was expressed at head and heart regions. **(F)** No fluorescent signal was detected in sagittal sections of E10.5 *Wdr60* ^*PB/PB*^ embryos. (N = 3 embryos from three litters for **(E, F)** IgG control was included. DAPI (blue) shows the nucleus. Scale bars: 1,000 µm. **(G)** Schematic *Wdr34* gene and transcript with PB insertion. **(H)** Transcriptional expression levels of *Wdr34* and the nearby genes *Sptan1* and *Set1* were measured by qRT-PCR in *Wdr34* ^*PB/PB*^ and WT (*Wdr34* ^*+/+*^) littermates. (*N* = 4 litters, at least 2 embryos per genotype were used in each litter) Statistical significance in (**B, D, H)** was calculated by two-sided unpaired *t*-test. *p < 0.05, **p < 0.01, and ns (not significant).

*Wdr60* and *Wdr34* genes were disrupted by piggyBac (PB) transposon system in mice respectively. As shown in Figure 1A, a DNA fragment containing RFP sequence was inserted between exon 4 and 5 of *Wdr60* gene. We firstly tested the efficiency of PB insertion in *Wdr60* ^*PB/PB*^ mice. There are four predicted transcriptional variants for *Wdr60*, which are indicated as transcript X1, X2, NM, and X5 in Figure 1A. The transcript NM (NM_146,039.3) is the functional *Wdr60* transcript, and it was significantly downregulated by PB insertion in *Wdr60* ^*PB/PB*^ homozygotes without affecting the neighboring genes *Esyt2* and *Vpr2* (Figure 1B). The protein encoded by this functional transcript NM is around 100 kDa in mice. To confirm the decrease of WDR60 protein, we extracted total protein from E14.5 heads and immunoblotted with anti-WDR60 antibody. As shown in Figures 1C, D, the band at 100 kDa was significantly decreased in *Wdr60* ^*PB/PB*^ mice compared to that in heterozygote (HET) mice. We also noticed a decrease of the 75 kDa band in the blot (Figure 1C). Therefore, we performed mass spectrometry on both 100 and 75 kDa bands. WDR60 is one of the proteins detected by mass spectrometry at 100kDa, while none of the 75 kDa proteins detected by mass spectrometry is WDR60 (Supplementary Table S1). Although other alternative transcripts of *Wdr60* was upregulated in *Wdr60* ^*PB/PB*^ mice (Figure 1B), they are probably not translated as no other protein isoform was detected. Immunofluorescent experiment was performed on E10.5 wild type (WT) embryo with anti-WDR60 antibody, and the results revealed that WDR60 is mostly expressed at the brain and heart regions at E10.5 (Figure 1E). Meanwhile, no fluorescent signal can be detected in *Wdr60* ^*PB/PB*^ mice with anti-WDR60 antibody (Figure 1F). Therefore, the expression of WDR60 was efficiently disrupted by PB insertion in *Wdr60* ^*PB/PB*^ mice.

The gene structure of *Wdr34* is relatively simpler and has only one transcript. *Wdr34* gene was interrupted by PB insertion between exon 1 and 2 (Figure 1G). The expression of *Wdr34* transcript is also significantly downregulated in *Wdr34* ^*PB/PB*^ mice without affecting the neighboring genes (Figure 1H). Hence, both *Wdr60* and *Wdr34* are knocked down efficiently in our models.

### Disruption of WDR60 provokes head malformation and heart defect

**FIGURE 2:**
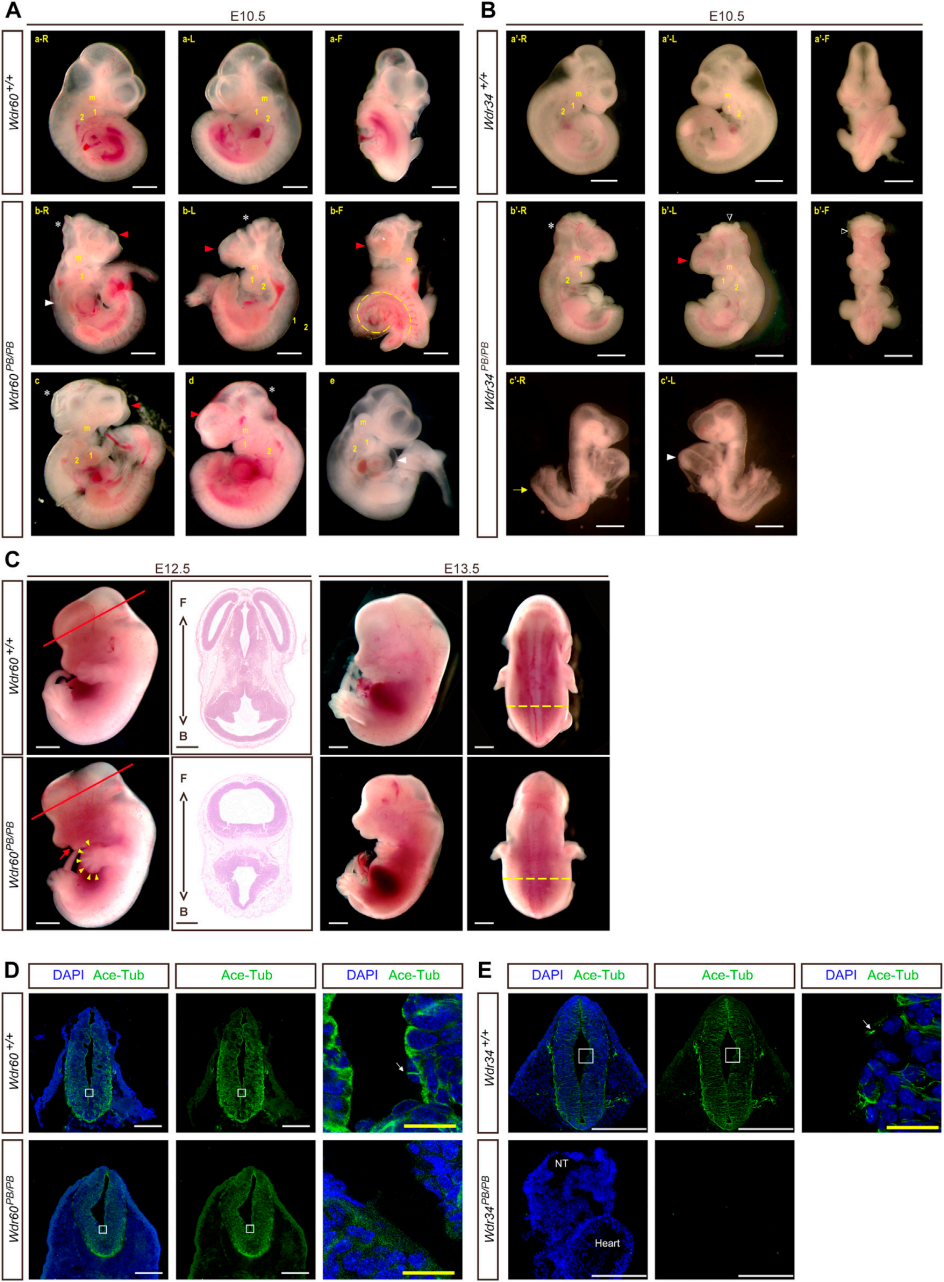
Deficiency of WDR60 and WDR34 elicits various developmental defects. Phenotypes of *Wdr60* ^*PB/PB*^ embryos **(A)** and *Wdr34* ^*PB/PB*^ embryos **(B)** at E10.5. a and a’ are WT embryos at E10.5. b-e are *Wdr60* ^*PB/PB*^ embryos at E10.5 b’ & c’ are *Wdr34* ^*PB/PB*^ embryos at E10.5. L, left side view; R, right side view; F, front view of each embryo. Read arrow heads indicate smaller telencephalic evagination. White stars mark the small midbrain. Arrowheads in white mark the defective heart, including cardiac edema and enlarged heart loop. Yellow dashed line depicts twisted tail. White triangles indicate the open head. Yellow arrow marks the open caudal neural tube. m, maxillary processes; 1&2, branchial arches. **(C)** Defective phenotypes of *Wdr60* ^*PB/PB*^ at E12.5 and E13.5. Read arrow indicates smaller lower jaw. Arrowheads in yellow indicate polydactyly. HE staining on the cross sections at the E12.5 embryo head (cross planes are indicated by red lines) showed the holoprosencephaly in *Wdr60* ^*PB/PB*^ mice. Yellow dashed lines showed broadened back by edema in E13.5 *Wdr60* ^*PB/PB*^. F, front. B, back. Immunofluorescent staining with anti-acetyl-Tubulin were performed on E10.5 transverse sections for *Wdr60* ^*PB/PB*^ and control embryos **(D)** and *Wdr34* ^*PB/PB*^ and control embryos **(E)**. The enlarged window were presented on the third panel. White arrows indicated cilia observed in the WT embryos. The acetyl-Tubulin was not detectable in *Wdr34* ^*PB/PB*^ embryo. NT, neural tube. All scale bars in white: 1000 µm. Scale bars in yellow: 100 µm.

Both *Wdr60* ^*+/PB*^ and *Wdr34* ^*+/PB*^ heterozygote mice appeared normal. Both *Wdr60* ^*PB/PB*^ and *Wdr34* ^*PB/PB*^ mice are embryonic lethality with a complete penetrance based on the results from heterozygotes breeding. Genotyping results of the offspring from heterozygotes breeding indicated that *Wdr60* ^*PB/PB*^ and *Wdr34* ^*PB/PB*^ embryos die at different stages (Tables 1, 2). *Wdr60* ^*PB/PB*^ mice die between E13.5 to E14.5 (Table 1), while *Wdr34* ^*PB/PB*^ mice die during E10.5 to E11.5 (Table 2 **)**. Most *Wdr60* ^*PB/PB*^ embryos exhibit abnormal head development at E10.5 with smaller telencephalic evagination and midbrain (Figure 2A, indicated by read arrow head and white star). The maxillary processes in *Wdr60* ^*PB/PB*^ embryos are more visible compared to that in WT embryos at E10.5 (Figure 2A). Some of the *Wdr60* ^*PB/PB*^ embryos also have cardiac edema and twisted tail at E10.5 (Figure 2A; Table 3). On the other hand, *Wdr34* ^*PB/PB*^ mice present severe developmental retardation and malformation at E10.5 (Figure 2B; Table 4). Similar to *Wdr60* ^*PB/PB*^ embryos at E10.5, some *Wdr34* ^*PB/PB*^ embryos at E10.5 also have smaller telencephalic evagination and midbrain (Figure 2B, indicated by read arrow head and white star). Consistent with the previous reported *Wdr34* null mice (Wu et al., 2017), some *Wdr34* ^*PB/PB*^ embryos at E10.5 also have open head (Figure 2B, white triangle). However, there are more *Wdr34* ^*PB/PB*^ embryos at E10.5 have severe growth retardation with an expanded heart loop and open caudal neural tube **(** Figure 2B; Table 4).

**TABLE 1:** Mendelian rations of *Wdr60* ^B/PB^ embryos at E10.5-14.5

Stage	Litters	Total	WT	HET	HOM	Dead	Absorbed
E10.5	12	107	17	63	27	0	2
E12.5	10	50	9	33	5	3 (HOM)	13
E14.5	12	51	7	45	2	4	6

**TABLE 2:** Mendelian rations of *Wdr34* ^PB/PB^ embryos at E10.5-13.5

Stage	Litters	Total	WT	HET	HOM	Dead	Absorbed
E10.5	8	53	14	27	12	0	11
E12.5	5	35	13	22	0	0	3
E13.5	4	31	9	22	0	0	10

**TABLE 3:** Phenotypes of *Wdr60* ^*PB/PB*^ embryos at E10.5 & E12.5

Stage	Total	Phenotype description		Count	Prevalence (%)
E10.5	27	abnormal	abnormal head development	25	92.6
			twisted tail	8	29.6
			heart edema	9	33.3
			growth retardation	1	3.7
		normal	no obvious defect	1	3.7
E12.5	8	abnormal	growth retardation (dead embryos)	3	37.5
			mandible defect	6	75.0
			twisted spine	2	25.0
			Polydactyly	2	25.0
			edema	3	37.5
		normal	no obvious defect	1	12.5

**TABLE 4:** Phenotypes of *Wdr34* ^PB/PB^ embryos at E10.5

Stage	Total	Phenotype description		Count	Prevalence (%)
E10.5	12	abnormal	abnormal head development	11	91.7
			growth retardation	10	83.3
			open head	3	25.0
			open caudal neural tube	8	66.7
			enlarged heart loop	10	83.3
		normal	no obvious defect	1	8.3

We were able to collect some *Wdr60* ^*PB/PB*^ embryos at E12.5 to E14.5. The ratio of total homozygotes at E12.5 still fit the Mendelian law. However, some of the homozygotes were dead at this stage. Therefore, the ratio of live homozygotes is less than expected at this stage (Table 1). The live *Wdr60* ^*PB/PB*^ embryos at E12.5 displayed multiple defects, including smaller lower jaw, polydactyl, and edema (Figure 2C). Transverse sections at the head of the E12.5 embryos revealed that *Wdr60* ^*PB/PB*^ embryos also have holoprosencephaly (Figure 2C).

We also performed immunofluorescent experiments on the transverse sections of E10.5 embryos with antibodies against acetyl-Tubulin, which is a cilia marker protein. High signal for acetyl-Tubulin can be observed in the neural tube, and cilia can be observed on the apical surface of the neural tube in WT embryos (Figures 2D, E). However, this signal was much less in the *Wdr60* ^*PB/PB*^ embryos (Figure 2D) and it was not detectable in the *Wdr34* ^*PB/PB*^ embryos (Figure 2E).

### WDR60 promotes SHH signaling and ventral neural fates commitment

**FIGURE 3:**
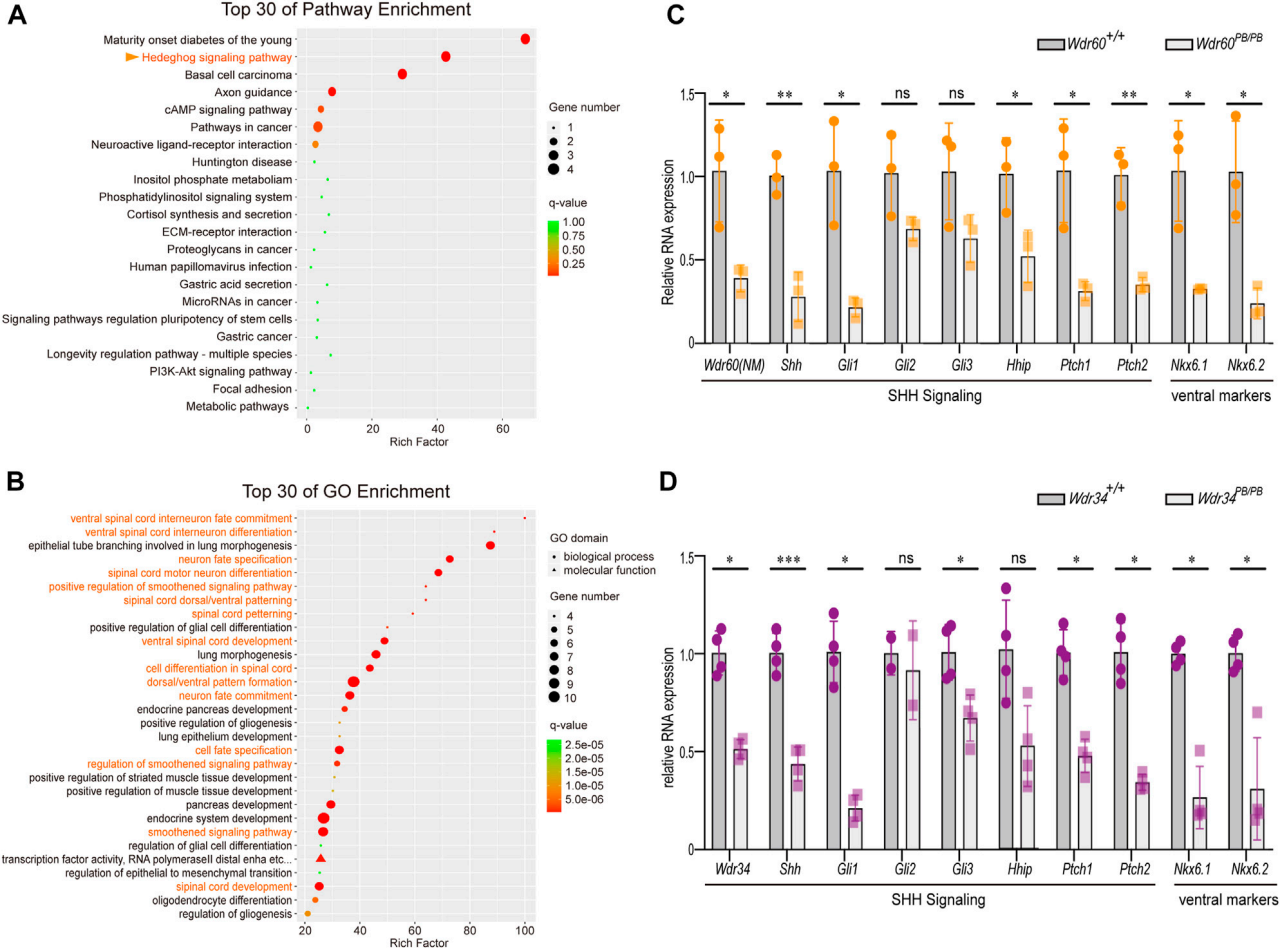
Deficiency of *WDR60* downregulates SHH signaling and ventral markers. Head tissues from E10.5 *Wdr60* ^*PB/PB*^ and WT embryos were sent for RNAseq. Differential expressive genes in *Wdr60* ^*PB/PB*^ and WT embryos were analyzed. **(A)** Top 30 enriched KEGG pathways and **(B)** top 30 GO enrichment terms were shown. Terms highlighted in orange are related to SHH signaling pathway and ventral neural tube development. The expression of key components of SHH signaling and ventral markers were confirmed by qRT-PCR in *Wdr60* ^*PB/PB*^ **(C)** or *Wdr34* ^*PB/PB*^ **(D)** and WT littermates. (*N* = 3 litters, at least 2 embryos per genotype were used in each litter) Statistical significance was calculated by two-sided unpaired *t*-test. *p < 0.05, **p < 0.01, ***p < 0.001, and ns (not significant).

**FIGURE 4:**
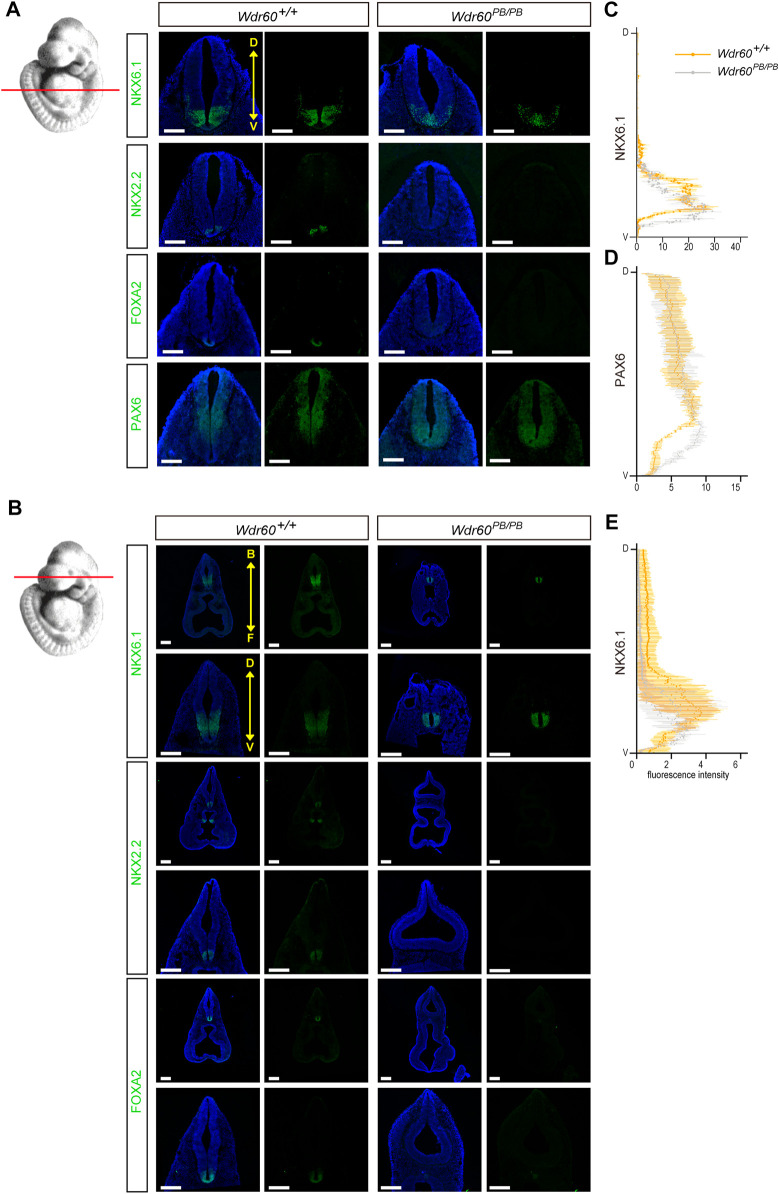
Deficiency of *WDR60* cause a dorsalized neural tube at E10.5. **(A, B)** E10.5 embryos from *Wdr60* ^*PB/PB*^ and WT embryos were sectioned transversely at two different cross planes, indicated by read lines on the embryo illustration at left. Immunofluorescent staining of neural tube pattern markers NKX6.1, NKX2.2, FOXA2, and PAX6 were performed on these sections. (*N* ≥ 3 embryos per genotype from at least three different litters) D, dorsal; V, ventral. B, back; F, front. DAPI (blue) shows the nucleus. All scale bars: 200 µm. The line profile plots for NKX6.1 **(C)** and PAX6 **(D)** distribution at heart level and NKX6.1 at head level **(E)** in the neural tube were provided. Immunofluorescent intensity of each marker were measured and calculated by ImageJ software.

As WDR60 is highly expressed in the head at E10.5, we collected the head tissues of *Wdr60* ^*PB/PB*^ and WT embryos at E10.5 for RNAseq experiment (Supplementary Table S2). Differential expressed genes are filtered and enriched by KEGG pathway analysis and Gene Ontology (GO) analysis. The results showed that both Hedgehog signaling pathway and ventral neural tube patterning were significantly downregulated in *Wdr60* ^*PB/PB*^ mice (Figures 3A, B). The downregulated genes included *Shh*, *Gli1*, *Hhip*, *Patched1*, *Patched2*, *Foxa1*, *Foxa2*, *Nkx6.1*, *Nkx6.2* and *Nkx2.2* (Supplementary Table S2). We further confirmed the downregulation of *Shh*, *Gli1*, *Hhip*, *Patched1*, *Patched2*, *Nkx6.1*, and *Nkx6.2* in *Wdr60* ^*PB/PB*^ embryos by qRT-PCR experiments (Figure 3C). We also collected whole embryos from *Wdr34* ^*PB/PB*^ and WT littermates at E10.5 for RNAseq experiment (Supplementary Table S3). SHH signaling was also downregulated in *Wdr34* ^*PB/PB*^ embryos and the downregulation of *Shh*, *Gli1*, *Patched1*, *Patched2*, *Nkx6.1*, and *Nkx6.2* was also confirmed by qRT-PCR in *Wdr34* ^*PB/PB*^ embryos (Figure 3D). Therefore, both WDR60 and WDR34 are positive regulators in SHH signaling. SHH signaling is important for ventral neural tube patterning and it has been reported that *Wdr34* ^*−/−*^ mice have dorsalized neural tube (Wu et al., 2017). We next examined the D-V neural tube patterning in *Wdr60* ^*PB/PB*^ embryos by immunofluorescent experiment. The results showed that the neural tube patterning is also abnormal in *Wdr60* ^*PB/PB*^ mice (Figure 4)*.* The most ventral domains were not differentiated as neither NKX2.2 nor FOXA2 were detectable in *Wdr60* ^*PB/PB*^ mice at two different transverse plains. The expression of NKX6.1 was still detectable in the *Wdr60* ^*PB/PB*^ neural tube, but the positive region extended towards the floor plate in *Wdr60* ^*PB/PB*^ mice at the heart level (Figures 4A–C, E). PAX6 positive region at this plane also extended to the floor plate in *Wdr60* ^*PB/PB*^ mice (Figures 4A, D).

### WDR34 has more effects on PCP pathway than WDR60

**FIGURE 5:**
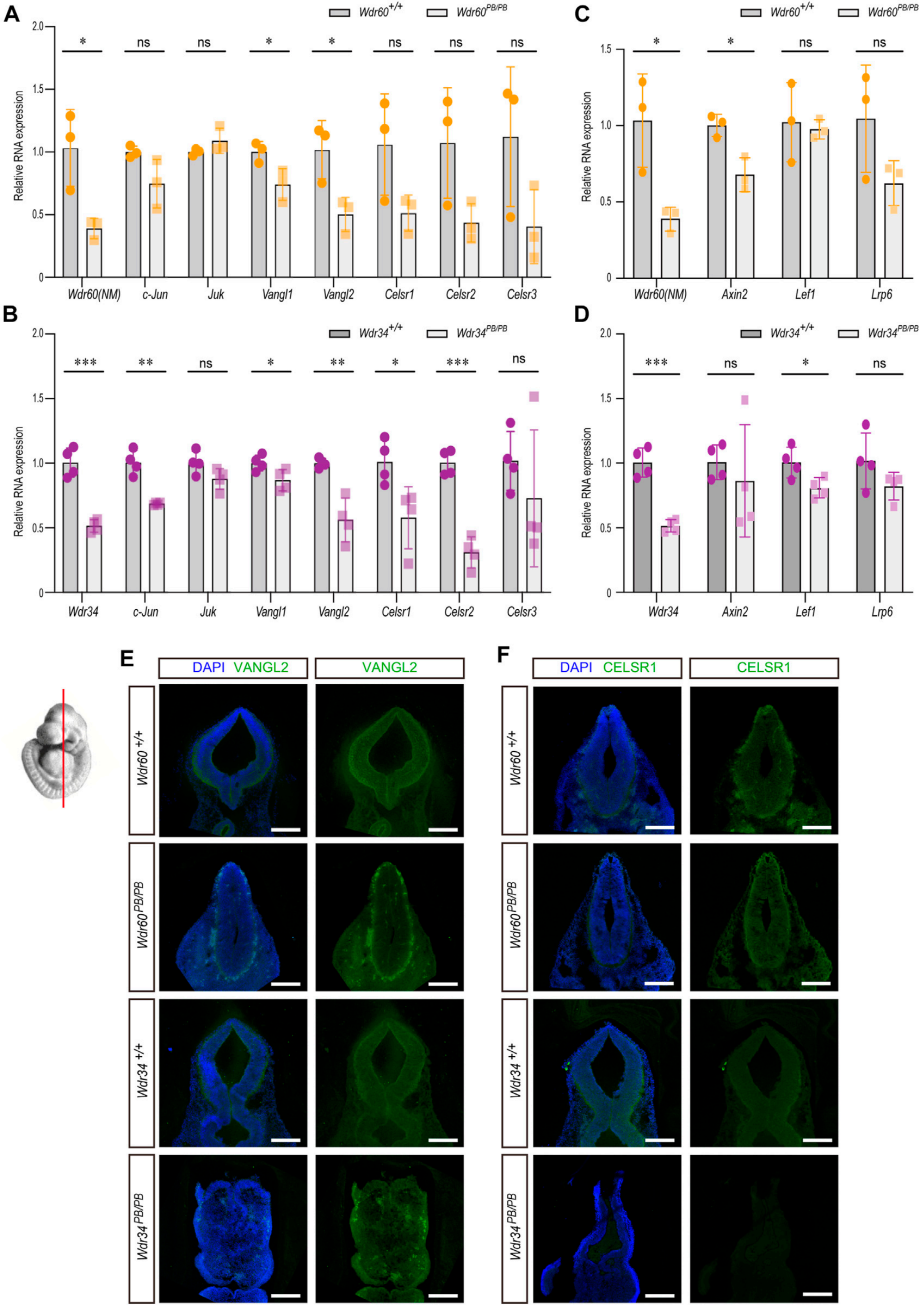
The different effects of WDR34 and WDR60 on PCP and WNT signaling. The expression of downstream targets and key components of PCP or WNT signaling pathway were detected by qRT-PCR in **(A, C)** *Wdr60* ^*PB/PB*^ and WT littermates or **(B, D)** *Wdr34* ^*PB/PB*^ and WT littermates. (N ≥ 3 litters, at least 2 embryos per genotype were used in each litter). Statistical significance was calculated by two-sided unpaired *t*-test. *p < 0.05, **p < 0.01, ***p < 0.001, and ns (not significant). Immunofluorescent experiments with anti-VANGL2 **(E)** and anti-CELSR1 **(F)** were performed on coronal sections of two mouse strains at E10.5. The images at the mid-brain area were shown here. All scale bars: 1000 µm.

Both WDR60 and WDR34 are positive regulator in SHH signaling and both *Wdr60* ^*PB/PB*^ and *Wdr34* ^*PB/PB*^ embryos have ventral patterning defects in neural tube. However, this cannot explain the different penetrance of NTD phenotypes between *Wdr60* ^*PB/PB*^ and *Wdr34* ^*PB/PB*^ embryos as we observed much high ratio of open cranial and caudal neural tube in *Wdr34* ^*PB/PB*^ embryos than that in *Wdr60* ^*PB/PB*^ embryos (Tables 3, 4). The open neural tube phenotype is unlikely due to the defects in SHH signaling as it was downregulated in both *Wdr60* ^*PB/PB*^ and *Wdr34* ^*PB/PB*^ embryos. Previously, we have reported that WDR34 can regulated PCP signaling *in vitro* (Yin et al., 2020). PCP signaling has been reported to play essential role in neural tube closure through enhancing midline apical constriction and the convergent extension of neural tube (Nikolopoulou et al., 2017; Wang et al., 2019). Therefore, we examined the expression of *c-Jun* and *Juk*, two downstream targets of PCP signaling, in both *Wdr34* ^*PB/PB*^ and *Wdr60* ^*PB/PB*^ embryos by qRT-PCR. Our results showed that the expression of *c-Jun* is significantly downregulated in *Wdr34* ^*PB/PB*^ embryos but not in *Wdr60* ^*PB/PB*^ embryos (Figures 5A, B). In addition, we also examined several key components of PCP pathway in both *Wdr34* ^*PB/PB*^ and *Wdr60* ^*PB/PB*^ embryos. While the expression of *Vangl1* and *Vangl2* was downregulated in both homozygotes compared to WT embryos, the expression of *Celsr1* and *Celsr2* was only significantly downregulated in *Wdr34* ^*PB/PB*^ embryos but not in *Wdr60* ^*PB/PB*^ embryos (Figures 5A, B).

Further immunofluorescent experiments were performed on coronal sections of E10.5 embryos with antibodies against VANGL2 and CELSR1. The expression of VANGL2 was decreased in both *Wdr60* ^*PB/PB*^ and *Wdr34* ^*PB/PB*^ embryos compared to their WT littermates (Figure 5E). The expression of CELSR1 was not affected in *Wdr60* ^*PB/PB*^ embryos; but it were significantly less in *Wdr34* ^*PB/PB*^ embryos compared to their WT littermates (Figure 5F). These results correlate well with the results in qRT-PCR experiments.

We also examined the downstream targets of canonical WNT signaling in both *Wdr34* ^*PB/PB*^ and *Wdr60* ^*PB/PB*^ embryos by qRT-PCR. There was a small decrease on *Axin2* in *Wdr60* ^*PB/PB*^ embryos and on *Lef1* in *Wdr34* ^*PB/PB*^ embryos compared to their WT littermates (Figures 5C, D).

### WDR34 but not WDR60 interact with IFT140

**FIGURE 6:**
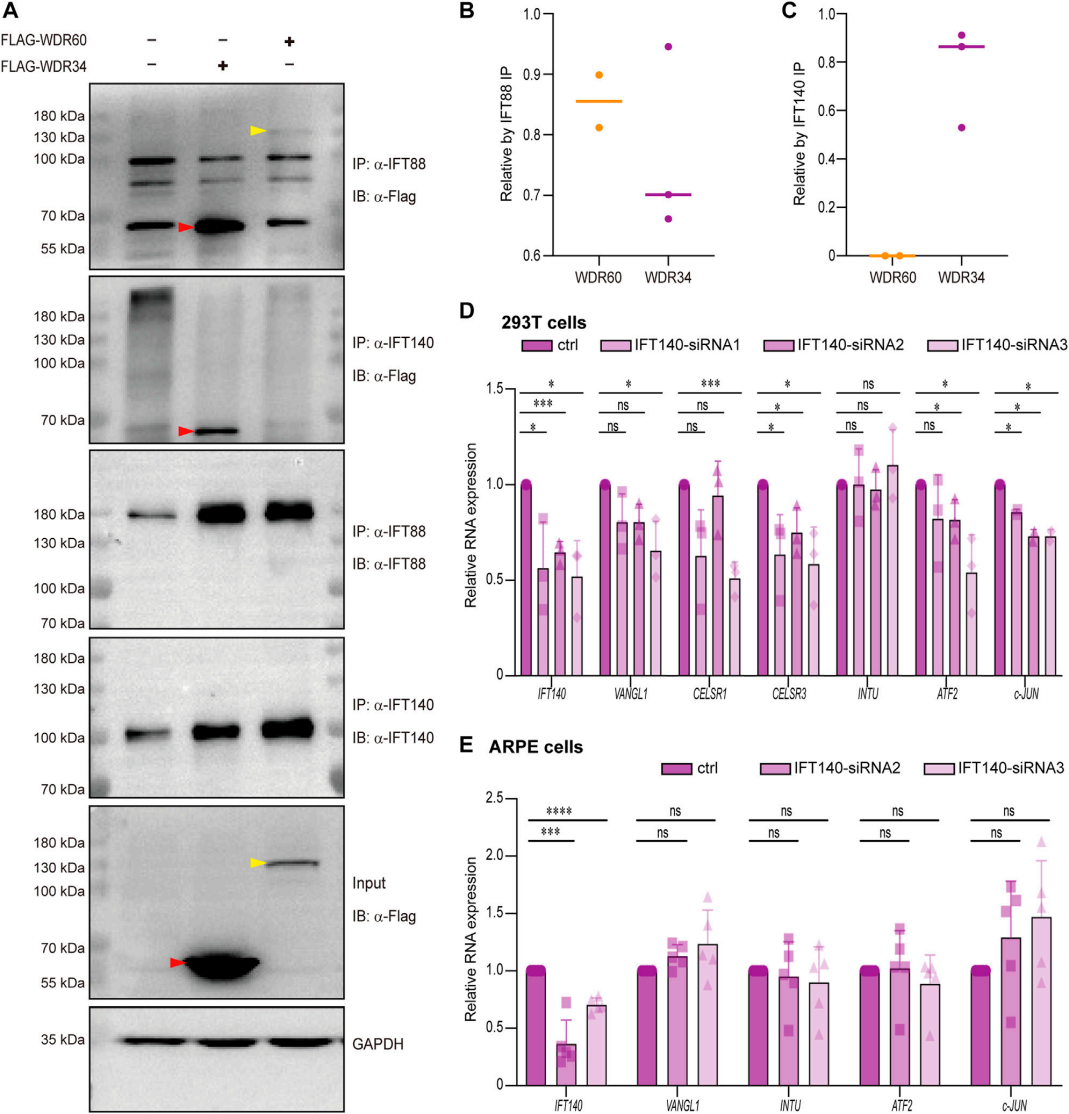
WDR34 but not WDR60 interacts with IFT140. **(A)** HEK-293T cells were transfected with either Flag-human WDR60 or Flag-human WDR34 plasmids. Co-IP experiments were performed by using anti-IFT88 or anti-IFT140 antibodies to pull down WDR60 and WDR34. Both WDR60 and WDR34 interact with IFT88, but only WDR34 not WDR60 interacts with IFT140 (n ≥ 4). The quantifications for relative WDR60 or WDR34 proteins pulled by either anti-IFT88 or anti-IFT140 were shown in **(B)** and **(C)** respectively. Human 293T cells **(D)** or human ARPE-19 cells **(E)** were transfected with siRNAs to knockdown *IFT140*. The expression of key components of PCP signaling pathway were detected by qRT-PCR. N ≥ 4. Statistical significance was test by two-sided unpaired *t*-test. *p < 0.05, **p < 0.01, ***p < 0.001, ****p < 0.0001 and ns (not significant).

Previous study on cells have revealed that WDR60 and WDR34 have some distinctive functions in cilia as WDR60 but not WDR34 is responsible for dynein-2 assembly, whereas WDR34 but not WDR60 is essential for axonemal extension (Vuolo et al., 2018). We noticed that in that study, knockout of *WDR60* or *WDR34* seem to have different effect on IFT140 disruption in cilia as IFT140 was stuck at the cilia tip in *Wdr34* knockout cells, but still remained at ciliary median shaft in *Wdr60* knockout cells (Vuolo et al., 2018). It has been reported that *Ift140* mutant mice also exhibit open brain phenotypes (Miller et al., 2013). Therefore, we hypothesized that IFT140 may be one of the key components different between WDR60 and WDR34. We first performed co-IP experiments to test whether WDR60 and WDR34 have different affinity with IFT140.293T cells were transfected with flag-tagged human WDR60 or WDR34 plasmids. IFT140 or IFT88 were pulled down by their specific antibody, and WDR60 or WDR34 pulled down together with IFT proteins were detected by western blot. Our co-IP results revealed that indeed only WDR34 but not WDR60 interact with IFT140 (Figures 6A, C). At the same time, both WDR60 and WDR34 interact with IFT88 (Figures 6A, B), which correlates with the similar distribution defects of IFT88 in *Wdr60* and *Wdr34* knockout cells (Vuolo et al., 2018). Since *Ift140* mutant mice also exhibit open brain phenotypes (Miller et al., 2013), it is possible that IFT140 is involved in PCP signaling as well. Therefore, we tested whether IFT140 can regulate PCP signaling in human ARPE-19 and 293T cells. SiRNAs for *IFT140* was transfected into 293T or ARPE-19cells and the expression of PCP downstream targets *c-JUN*, *INTU*, *ATF2* and PCP pathway components *VANGL1* and *CELSR1* were detected by qRT-PCR. Our results showed that knockdown of *IFT140* could downregulate PCP signaling in 293T cells as the expression levels of *c-JUN* and *ATF2* were significantly downregulated in *IFT140* knockdown cells (Figure 6D). However, no effect could be observed in ARPE-19 cells (Figure 6E).

## Discussion

In this study, we uncovered WDR60 and WDR34, identified as the heterodimers of dynein-2 intermediate chain, exert both collective and separate functions during embryogenesis. Both WDR60 and WDR34 can promote SHH signaling and involved in neural tube ventral patterning. However, there are distinctive differences between *Wdr60* ^*PB/PB*^ and *Wdr34* ^*PB/PB*^ embryos. First of all, deficiency of WDR60 or WDR34 led to different timing of embryonic lethality. Secondly, the NTD phenotypes are quite different between *Wdr60* ^*PB/PB*^ and *Wdr34* ^*PB/PB*^ homozygotes. Although *Wdr60* ^*PB/PB*^ embryos often have a twisted tail, the neural tube is closed; while *Wdr34* ^*PB/PB*^ embryos have much high prevalence of open neural tube in both the cranial and caudal regions. We further proved that WDR34 has more effects on PCP pathway components. In addition, WDR34 but not WDR60 is interacted with IFT140.

The holoprosencephaly phenotype and spinal cord patterning changes in *Wdr60* ^*PB/PB*^ mice consistent with impaired Shh signaling. Indeed, the expression of SHH signaling down-stream targets (*Gli1*, *Hhip*, *Ptch1*, and *Ptch2*) and ventral neural tube patterning markers (*Foxa2*, *Nkx2.2*, *Nkx6.1*, and *Nkx6.2*) were downregulated in *Wdr60* ^*PB/PB*^ mice compared to that in WT littermates. We also confirmed the downregulation of ventral markers NKX6.1, NKX2.2, and FOXA2 through immunostaining experiments. SHH signaling is also downregulated in *Wdr34* ^*PB/PB*^ mice. Therefore, WDR60 and WDR34 probably function together in regulating SHH signaling.

However, these two homozygotes die at different stages and *Wdr34* ^*PB/PB*^ mice have much higher prevalence of open neural tube at E10.5. Such open neural tube phenotype is unlike due to lack of SHH signaling as normally deficiency on the negative regulators of SHH signaling lead to open neural tube phenotypes in mice (Murdoch and Copp, 2010). Previously, we have reported that a human NTD mutation in WDR34 and we demonstrated that WDR34 can regulated PCP signaling *in vitro* (Yin et al., 2020). Here, by using mouse models, we also observed that the expression levels of c-Jun and CELSR1&2 were downregulated in *Wdr34* ^*PB/PB*^ mice but not in *Wdr60* ^*PB/PB*^ mice. We also found that only WDR34 interact with IFT140 but not WDR60. This difference in the interaction correlates well with the previous discovery WDR34 having greater impact on IFT140 ciliary localization than WDR60 (Vuolo et al., 2018). What’s more, the open brain phenotype in *Ift140* mutant mice (Miller et al., 2013) is also similar to that in our *Wdr34* ^*PB/PB*^ mice and the reported *Wdr34* null mice (Wu et al., 2017). It is possible that WDR34 may regulate PCP signaling through IFT140. However, further experiments will be needed as we only observed downregulation effect on PCP signaling by knocking down IFT140 in 293T cells but not ARPE cells. More cells with or without cilia are needed to further explore the function of WDR34 and IFT140 in regulating PCP signaling.

The role of IFT proteins and cilia in PCP signaling has been controversial. Couple of reports have showed that IFT88 and cilia are not required to establish PCP in zebrafish embryos (Borovina and Ciruna, 2013) and fibre cells in mouse lens (Sugiyama et al., 2016). On the other hand, there are studies showed that cilia proteins such as IFT20 (May-Simera et al., 2015) and ICK (Okamoto et al., 2017) are upstream of PCP asymmetry in cochlea. To be noticed, both IFT88 and IFT20 are IFT-B subunits. In our study, we also did not observe any different interaction of WDR34 and WDR60 on IFT88. It was IFT140, the IFT-A subunit protein has different interaction with WDR34 and WDR60. Besides IFT140, mouse mutants of several other IFT-A subunits, such as IFT144 (Ashe et al., 2012), IFT122 (Cortellino et al., 2009; Qin et al., 2011), and IFT139 (also called Ttc21b) (Stottmann et al., 2009; Brooks et al., 2020), all have exencephaly phenotype; along with that of the IFT-A adapter TULP3 (Norman et al., 2009; Patterson et al., 2009), and IFT-A/TULP3 cargo GPR161 (Mukhopadhyay et al., 2013). Whether WDR34 and/or WDR60 interact with other IFT-A subunits and how they regulate neural tube development together will be worth studying in the future.

Another interesting observation is that both WDR34 and WDR60 deficiency can affect the expression of PCP core components and WDR34 has stronger effects. It has been reported that PCP can regulate ciliogenesis (Park et al., 2006; Gray et al., 2009; Heydeck et al., 2009; Kim et al., 2010; Zeng et al., 2010). However, very little is known about whether cilia can regulate PCP components. How these two cilia proteins WDR34 and WDR60 regulate PCP components and why they have different effects on different components will need further investigation. It has been known that defects in transcription factors such as Med12 (Rocha et al., 2010), CDX (Savory et al., 2011), and Zic3 (Bellchambers and Ware, 2021) can reduce the expression of PCP components and cause severe NTDs in mouse models. Zic3 deficiency also leads to abnormal cilia position and left-right signaling in mouse (Bellchambers and Ware, 2021). Further comparation studies should be carried out to understand the functions of these cilia proteins in regulating PCP components.

Although we focused on neural phenotypes caused by WDR60 and WDR34 deficiency, we also observed that WDR60 is involved in cardiac development. First of all, WDR60 is highly expressed in the heart at E10.5 (Figure 1E). Secondly, deficiency of WDR60 results in cardiac edema. Random dextrocardia was also found in *Wdr60* ^*PB/PB*^ embryos, which is a typical ciliopathy phenotype. Therefore, what is the function of WDR60 in cardiac development and whether defects/mutations in this gene is associated with congenital heart defects will be worth for further investigation.

## Materials and methods

### Mouse stains

The mice used for experiments were housed at the Animal center of the Institute of Developmental Biology and Molecular Medicine, Fudan University. FVB-*Wdr60* ^*PB/+*^ and C57BL-*Wdr34* ^*PB/+*^ strains were generated based on previously reported method (Ding et al., 2005). In brief, mice carrying a single PB transposon PB [Act-RFP] in the genome was mated with the transposase line Act-PBase (Ding et al., 2005), which were generated by conventional pronuclei injection of linear plasmids. Double-positive male progeny were then crossed with WT mice to generate transposase negative mutants carrying remobilized PB insertions. The *Wdr60* and *Wdr34* mutations were one of the mutagenesis lines generated in the offspring. The mutation was mapped by inverse polymerase chain reaction (PCR) and with the primers listed below. *Wdr60* mutant carries the PB insertion in the third intron of the *Wdr60* gene (Chr12:116,218,415 bp, Ensemble 109). *Wdr34* mutant carries the PB insertion in the first intron of the *Wdr34* gene (Chr2:29932629 bp, Ensemble 109).

The primers for genotyping *Wdr60* are:

PB: 5′-CTG​AGA​TGT​CCT​AAA​TGC​ACA​GCG-3′,

GL: 5′-TCA​GAG​GTA​GTC​TTT​GCC​CAC​C-3′, and

GR: 5′-CCC​AAG​CTG​CTT​GTT​AGT​TTG​C-3’.

The primers for genotyping *Wdr34* are:

PB: 5′-CTG​AGA​TGT​CCT​AAA​TGC​ACA-3′,

GL: 5′-GCT​ACA​TTT​CTG​GTT​AGT​CTG​GGT​C-3′, and

GR: 5′-TGT​CTA​GCA​CGG​CAC​ATG​CA-3’.

Images of mouse embryos were captured on Leica stereoscopic microscope. All animal experiments were carried out in strict accordance with the recommendations in the Guide for the Care and Use of Laboratory Animals of Fudan University. The protocol was approved by the Committee on the Ethics of Animal Experiments of Fudan University.

### Plasmids

Human *WDR60* and *WDR34* cDNA were cloned into pCMS-EGFP vector and the plasmids were generously provided by Prof. Zhiheng Xu of Chinese Academy of Science. The primers used for cloning WDR60 were 60-F-XhoI: 5′-CCG​CTC​GAG​ATG​GAG​CCC​GGG​AAG​A-3′ and 60-R-KpnI: 5′-AAT​TCT​AGA​TCA​GGC​CGC​CAC​CTC​TGC-3’. The primers used for cloning WDR34 were 34-F-EcoRI: 5′-ATT​AGA​ATT​CCA​TGG​CAA​CCC​GCG​CG-3′ and 34-R-XbaI: 5′-AAT​TCT​AGA​TCA​GGC​CGC​CAC​CTC​TGC-3’.

### Cell culture

HEK-293T cells were grown in high-glucose DMEM or DMEM/F12 supplemented with 10% FBS and 1% penicillin/streptomycin in a 5% CO_2_ incubator at 37°C. Human adult retinal pigmented epithelium-19 (ARPE-19) cells were grown in DMEM/F12 supplemented with 10% FBS and 1% penicillin/streptomycin in a 5% CO_2_ incubator at 37°C. Transfection was performed using FuGENE^®^ HD Transfection Reagent (Promega) according to the manufacture’s instruction.

### RNA sequencing

The head tissue of E10.5 mice were dissected and collected on ice. Total RNA of samples was extracted and fragmented for building cDNA libraries. The libraries were quality controlled and were sequenced on the Illumina HiSeq^®^ 2,500 System. The differential expressive genes were sorted by fold change >2 plus q-value <0.05, and were analyzed by KEGG pathway enrichment and Gene Ontology (GO) enrichment.

### qRT-PCR

Embryos at stages were dissected on ice and oolemma of each individual was kept for genotyping. Total RNA was extracted with TRIzol reagent (Invitrogen) and chloroform, precipitated with isopropanol, washed by 70% RNase-free ethanol and reverse transcribed into cDNA. qRT-PCR experiments were performed using SYBR^®^ Green Realtime PCR Master Mix (Code No. QPK-201) on the LightCycler^®^ 480 Real-Time PCR System (Roche). Relative expression of each target was calculated with mouse *Gapdh* as control (Primers listed in Supplementary Table S4).

293T and ARPE-19 cells transfected with siRNAs were also lysed and total RNA was extracted as described above. Human *GAPDH* was used as internal control.

The sequences for siRNAs for IFT140 are as follow: siRNA-1 5′-UAA​ACA​CCG​UCA​CUU​CUC​CTT-3′, siRNA-2 5′-UAA​UAA​GGC​ACU​UCC​AUC​CTT-3′, siRNA-3 5′-AUG​UUC​UUC​AUG​AUC​UCC​GTT-3’.

### Immunofluorescence and microscopy

E10.5 Embryos were fixed in 4% Paraformaldehyde at 4°C overnight, equilibrated in 1xPBS/15% sucrose and 1xPBS/30% sucrose sequentially till they sink to the bottom and then were embedded in OCT. Embedded embryos were sectioned with 20 µm thickness per section. Embryo section slides were washed in 1xPBS solution to remove OCT, then fixed in 4% Paraformaldehyde for 10 min, washed in 1xPBS for 3 × 3 min and permeabilized in 1xPBS/0.5% Triton-X 100 at 4°C for 2 h. Permeabilized sections were blocked in 1xPBS/0.05% Triton-X 100 with 5% goat serum for 2 h, incubated in primary antibodies at 4°C overnight, incubated in secondary antibodies avoiding light at room temperature for 2 h (antibodies listed in Supplementary Table S5). Then sections were immersed in 1xPBS/DAPI for 10 min, rinsed in 1xPBS 3 × 3 min and sealed for further imagingImmunofluorescence images of embryonic sections were taken with a Zeiss LSM 700 confocal microscope.

### Immunoblotting and immunoprecipitation

For immunoblotting, E10.5 mouse embryo tissues were lysed in pre-chilled 1% Triton X-100 buffer (20 mM Tris buffer at pH7.5, with 150 mM NaCl plus sodium pyrophosphate, *β*-glycerophosphate, EDTA, Na_3_VO_4_, and leupeptin) for 10 min on ice. Protein lysates were collected after high-speed centrifugation and mixed with 5x SDS-PAGE loading buffer and denatured at 95°C for 5 min. Denatured proteins were separated by SDS-PAGE, electro-transferred to PVDF membrane and probed with primary and secondary antibodies (antibodies used in Supplementary Table S5). Blot images were captured on Tanon™ 5200CE Chemi-Image System and analyzed in ImageJ.

For immunoprecipitation, protein lysates from 293T cells were prepared as above and incubated in 1xPBS/IP antibodies on rotator at 4°C overnight. Then protein solutions were incubation with antibody and Protein A + G Agarose beads on rotator at 4°C for 2 h. Agarose beads were washed in cold 1xPBS and collected by 2,500 rpm centrifugation for three times. Proteins were eluted from beads with 5x SDS-PAGE loading buffer and denatured at 95°C for 5 min. Denatured immunoprecipitants were acquired by high-speed centrifugation, and analyzed by electrophoresis as described in immunoblotting.

### Mass spectrometry

Protein lysates were gained and separated by SDS-PAGE as described above. Whole SDS-PAGE gel was stained by Coomassie brilliant blue. Stained gel zones parallel to 100 kDa and 75 kDa markers were cut out for Mass spectrometry analysis. Mass spectrometry experiments were performed at the State Key Laboratory of Genetic Engineering Fudan University.

## Data Availability

The datasets presented in this study can be found in NCBI online repositories. The accession number(s) are: Bio Project accession number: PRJNA900366. The link is https://www.ncbi.nlm.nih.gov/bioproject/PRJNA900366.
