# Supplementary material for: Deficiency of Wdr60 and Wdr34 cause distinct neural tube malformation phenotypes in early embryos
Source: Front Cell Dev Biol. 2023 May 9;11:1084245. doi: 10.3389/fcell.2023.1084245 (PMC10203710; doi:10.3389/fcell.2023.1084245)
Supplement: Supplementary file 3 [file Table1.DOCX]

**Table S1.**

**WD-repeat proteins verified by Mass spectrometry in E14.5 mice heads**

| **Molecular mass** | **Accession** | **Target Proteins** |
| --- | --- | --- |
| 100 kDa | Q99ME2 | WDR6 |
|  | G5E8J3 | WDR11 |
|  | O88342 | WDR1 |
|  | Q8CGF6 | WDR47 |
|  | Q8K4P0 | WDR33 |
|  | **Q8C761** | **WDR60** |
|  | Q8BHB4 | WDR3 |
| 75 kDa | Q8BH57 | WDR48 |
|  | O88342 | WDR1 |
|  | A0A3Q4EHN7 | WDR43 |
|  | E0CYH4 | WDR26 |
|  | Q9Z0H1 | WDR46 |
|  | F6RAR9 | WDR86 |
|  | Q3TWF6 | WDR70 |
|  | S4R1X1 | WDR91 |
