# Supplementary material for: Deficiency of Wdr60 and Wdr34 cause distinct neural tube malformation phenotypes in early embryos
Source: Front Cell Dev Biol. 2023 May 9;11:1084245. doi: 10.3389/fcell.2023.1084245 (PMC10203710; doi:10.3389/fcell.2023.1084245)
Supplement: Supplementary file 4 [file Table5.DOCX]

**Table S5. Antibodies information**

| **Antibodies** | **ID # (Company)** | **Dilution** |
| --- | --- | --- |
| Anti-FOXA2 | ab108422 (Abcam) | IF (1:1000) |
| Anti-Nkx2.2 | ab191077 (Abcam) | IF (1:900) |
| Anti-NKX6.1 | ab221549 (Abcam) | IF (1:300) |
| Anti-PAX6 | 12323-1-AP (Proteintech) | IF (1:400) |
| Anti-WDR60 | HPA020607 (Sigma-Aldrich) | IF (1:50); WB (1:1000) |
| Anti-alpha Tubulin (acetyl K40) | ab24610 (Abcam) | IF (1:500) |
| Anti-VANGL2 | 21492-1-AP (Proteintech) | IF (1:100) |
| Anti-CELSR1 | LS-C119447 (LifeSpan BioSciences) | IF (1:200) |
| Anti-IFT140 | 17460-1-AP (Proteintech) | Co-IP (1:100) |
| Anti-IFT88 | 13967-1-AP (Proteintech) | Co-IP (1:100) |
| Anti-DYKDDDDK (FLAG®) tag Mouse mAb | AT0022 (Engibody) | WB (1:1000) |
| Anti-GAPDH Mouse mAb | AT0002 (Engibody) | WB (1:1000) |
| Goat Anti-Rabbit IgG H&L (Alexa Fluor® 488) | ab150081 (Abcam) | IF (1:1000) |
| HRP-labeled Goat Anti-Rabbit IgG(H+L) | A0208 (Beyotime) | WB (1:1000) |
| HRP-labeled Goat Anti-Mouse IgG(H+L) | A0216 (Beyotime) | WB (1:1000) |
