# Supplementary material for: Deficiency of Wdr60 and Wdr34 cause distinct neural tube malformation phenotypes in early embryos
Source: Front Cell Dev Biol. 2023 May 9;11:1084245. doi: 10.3389/fcell.2023.1084245 (PMC10203710; doi:10.3389/fcell.2023.1084245)
Supplement: Supplementary file 5 [file Table4.DOCX]

**Table S4. Primers used in qRT-PCR**

| **Targeted Gene** | **Forward-primer** | **Reverse-primer** |
| --- | --- | --- |
| *Wdr60 (NM)* | GAGCCTGGGAAGAGAAGAACCAAAG | CGAGGATGTTCTGTGGAGGTG |
| *Wdr60 (NM, X1, X2)* | GCCCTCAAGCAAAAGACACG | CTTCTCCTGGGTGTTGGGTC |
| *Wdr60 (NM, X1, X2, X5)* | GGCACCGACATGGGACTTAT | CACCGTGTTGCTCAGGTTTG |
| *Esyt2* | GGGCTTCTTGGCAAAGTGCT | TCCATGTTGAGGTGCCACG |
| *Vipr2* | AACGGAGAATCAGAGAGCCTG | ACCATCCATCGCTAGTGCAG |
| *Wdr34* | TGGCAGAGTCATGCCTTCGATG | CTGTCGAGTTCCAGGAAATGCC |
| *Sptan1* | GGCACTTTCCAGGCATTTGAG | GGCAGTGGTCCAGCATCAT |
| *Set1* | AGAAGAGGTCAGAATTGATCGCC | TGGTTGACAAATGTTGTTACCCA |
| *Gli1* | CTCAAACTGCCCAGCTTAACCC | TGCGGCTGACTGTGTAAGCAGA |
| *Gli2* | ACACTGTGGAGGACTGCCTACA | GGCATCTCCATGCCACTGTCAT |
| *Gli3* | CTGCGGTATCTCCTCTCATAGG | CAGCACTGTGAAGTCTACACCTG |
| *Hhip* | GAAACGGCTACTACACCCCC | TGGCTCACACTTGGCAATTC |
| *Ptch1* | CCTGGCAGAGGACTTACGTG | AGGCATAGGCAAGCATCAGT |
| *Ptch2* | CCTTTGCCCCTGTGACTGAT | TGTATACCTGCACCACCTGT |
| *Nkx6.1* | CTGGACAGCAAATCTTCGCC | TCTGGAACCAGACCTTGACCT |
| *Nkx6.2* | CGAGAGCCAAGTGAAGGTGT | TGTGCTTTTTGAGAAGCCGC |
| *c-Jun* | CCTTCTACGACGATGCCCTC | GGTTCAAGGTCATGCTCTGTTT |
| *Juk* | ATGGCTGTCGATATTCAACCAG | CCTCTTGGGCATACCCCAC |
| *Vangl1* | CACGGCAGCAGCACTACCAC | CCATCCCGTAACCCGTTTGT |
| *Vangl2* | GGGATGGGAGTCGTGGAGATA | TCATGGGAGATACTGTGCTCAG |
| *Celsr1* | CTCTTATTCTTGCCACCACT | GATTTCTACATTGAGCCCAC |
| *Celsr2* | TACATCCCCTTCTTGCTGAGG | GATGAGTGGGTGGAGGCATAG |
| *Celsr3* | TGGCCCTATCGTCCTCGTTA | CTTGCCTAGACAGGCTGGTG |
| *Gapdh* | TGGAGAAACCTGCCAAGTATG | GGAGACAACCTGGTCCTCAG |
| *IFT140* | GAGTGCGTGCCAGATACACA | CAAGACACCAAGCCTGTCCC |
| *VANGL1* | GACACAAGTCACCCCGGAATA | TCCTCTGTCCGAGTAGAATCATT |
| *VANGL2* | GCTCCCGATCTGATTCCTGA | TATTTTGCTGGACGGGTGGG |
| *CELSR3* | CCCACCCCAAAGATGTGGAT | GGCTGGTTGTTGTTAGCTGC |
| *INTU* | TCAGCGACTCGGGTTCAT | CAGCCATTCAGGCTCAAGA |
| *ATF2* | AATTGAGGAGCCTTCTGTTGTAG | CATCACTGGTAGTAGACTCTGGG |
| *GAPDH* | TCAAGGCTGAGAACGGGAAG | CGCCCCACTTGATTTTGGAG |
